# Ergot as an Anti-Hemorrhagic

**Published:** 1873-10

**Authors:** F. Curtis Smith

**Affiliations:** Middleport, O.


### SOUTHERN MEDICAL RECORD.

VOL. III.—OCTOBER, 1873.—NO. 10.

### PART I.

### ORIGINAL COMMUNICATIONS.

### ERGOT AS AN ANTI-HEMORRHAGIC.

BY F. CURTIS SMITH, OF MIDDLEPORT, O.

The oxytocic properties of ergot have been known as a domestic agent since the early part of the seventeenth century. In 1668 Camararius brought it before the profession, and Bautymanni in 1699. In 1774 its domestic use, and dangers from the same became so general in France as to induce legislative enactment, prohibiting its employment by midwives. After this it fell into general disuse until 1807, when Dr. Stearnes, of Saratoga, N. Y., and in 1813 Dr. Prescott, of the same State, brought it prominently before the profession. The agent thus came into general use, and its oxytocic roperties well known, and the proper conditions indicating its application well described by American writers and teachers, for a score of years before becoming generally adopted in England and Europe.

But while its power as a parturient was well known, it was left for later investigators to learn and prove its efficacy as an anti-hemorrhagic. Even in cases of uterine hemorrhage, I have been unable to find the least reference to its use until that of M. Goupil,

(Med. Chirug. Rev. vol. x, No. 27, p. 251), in 1827, who related a case of recent labor, where profuse hemorrhage occurred after the placenta had been delivered. "Cold applications, and the introduction of the hand into the uterus failed to arrest the flow of blood." He then gave ergot, which was followed in a few minutes by a severe and painful contraction of the uterus, causing the undue hemorrhage to cease. This must have been about the time of its introduction for uterine hemorrhage, as is indicated by further remarks in the same article above referred to.

From this date onward, it came gradually into use for various forms of uterine hemorrhage, and was mentioned by Churchill, Dewees, and other obstetricians that wrote during the fourth and fifth decade of the present century, as a valuable agent in controlling menorrhagia, metrorrhagia, and post partum hemorrhage. Since that time its use as an uterine anti-hemorrhagic have become too well known to require delineation here. Few, if any, doubt its power in controlling to a great extent, all forms of uterine hemorrhage, where there is not some extraneous cause for its continuance, and where surgical means are necessarily resorted to for the purpose of effecting the desired end.

The earliest mention I have found of its application as an anti-hemorrhagic in other organs, is that by Durante De Caserta, in probably the latter part of 1835, (Amer. Jour. Med. Sci. for Feb., 1836, p. 500), in case of sanguine temperament, a child aged 12 years, where a copious hemoptysis, and epistaxis followed the use of an emetic given for the relief of an attack of colic. M. Durant "ordered a copious venesection and iced drinks, acidulated with mineral acids." * * These remedies were successful, but on the following day the hemorrhage was renewed with much greater severity, and returned at every attack of coughing, alternating with epistaxis." He then gave five grains of powdered ergot every two hours, with the effect of permanently stopping the hemorrhage.

Waring in his work on therapeutics, refers to Mr. Ings, who used ergot as an anti-hemorrhagic in 1834, also to Pegnacea, Negri, and others, all of whom seem to have used it in atonic hemorrhage, hemoptysis, hematemesis, hematuria, and epistaxis. But even Waring, in the latter part of the last decade, thinks the statements of these authorities need further confirmation.

From 1835 to 1870 there is found occasional reference in medical literature to its power in checking the flow of blood from internal organs other than the uterus. Since the latter date its standing in this respect seems to have become pretty fairly established.

In The Practitioner for December, 1871, Dr. Currie Ritchie, of Manchester, Eng., relates nine cases of hemoptysis in which ergot or ergotine was used hypodermically, in eight of which the discharge of blood was promptly checked, and with a fair degree of permanency. In the one where the discharge was again observed, it was the result of imprudence on the part of the patient. True, some of these cases were only under observation a fortnight, but the prompt action of the agent seemed unmistakable. One tolerably valid objection to the hypodermic use of ergot is its liability to produce painful induration or pustule. This however, can count but little against it where prompt action is demanded at our hands.

Dr. Drasche, (Wiener. Med. Wochenschrift), of Vienna, has also published a series of cases in which ergotine was used as an hemostatic. The greater number of his cases were those of hemoptysis from tubercular disease of the lungs, in which he found it to act promptly and well; but it was also resorted to in cases of hematemesis and epistaxis, with good results. He states that it is as efficacious as it is an easily employed remedy. "In some cases its effects were so rapid and so remarkable that no doubt could possibly be held respecting its action." He deems it our most reliable hemostatic, and as an "invaluable means when other agents have been tried without effect, or when sudden and profuse hemorrhage calls for instant action."

Among the valuable contributions on this subject is one recently from F. E. Anstie, (practitioner for February, April and May, 1873.) For nearly three years he has been comparing its effects as an "arrestor of hemorrhage from the lungs in phthisis," with other agents more commonly in use as "gallic acid, acetate of lead, digitalis, turpentine and alum." In some of his cases these agents were first administered with the usual degree of success, viz: that of temporarily checking the hæmoptysis. But with the ergot administered per orum he usually obtained quick and permanent relief from the bleeding. In his hands as in that of others who had tried the agent. An occasional case was met with where the hemorrhage would return, but where it was more amenable to the use of ergot than to the other agents used. Anstie states his opinion as follows:

"I think we have now established the fact (a) of the direct action of ergot in the cases which I have recorded; (b) of its superiority in several of these cases to other styptics that had been tried; (c)

the probability, from physiological analogies, that ergot would act more universally as a checker of hæmoptysis than the routine remedies with which we are familiar; (d) also, that it is perfectly safe for the purpose in view, and in this respect is superior to digitalis, which it otherwise resembles a good deal."

My own experience with the agent as an arrestor of hemorrhage, other than uterine, is quite limited but very satisfactory as far as tried. In two cases of obstinate hæmaturia ergot permanently checked the discharge after plumbi acetas, tannin, per sulp. of iron, and opium had signally failed. No unpleasant symptoms resulted from its use. In a very obstinate and profuse case of hæmatemesis occurring four years since in a lady in Clifton, I had used all the agents above named with only very temporary effect. Later in the case menorrhagia set in, for which I gave very large doses of powdered ergot (ʒ ss to ʒ i every two hours,) as the discharge was profuse, and my patient quite exsanguinated. The first dose was rejected, and with it several ounces of dark lumpy blood. Another dose was immediately administered which checked the free menorrhagia, and, to my surprise, the stomachic hemorrhage also. The agent was continued in smaller doses for several days. The hæmatemesis failing to return ever afterwards. The menorrhagia, however, returned at the next molimen.

In a recent case of hemorrhage from the bowels I prescribed the fluid extract of ergot with apparently the happiest effects in securing relief. As an internal hæmostatic I have found nothing to compare with a combination of ergot and monsels salt. It has seldom failed for me when thus combined to arrest any bleeding that can be checked by the simple use of remedies by the stomach. In some cases, however, the iron will not be borne, while the ergot alone will be readily and easily retained. It will be asked how does this agent accomplish these effects. A question easier asked than answered satisfactorily. Anstie thinks it produces contraction of the smaller blood vessels, and also slows the heart, thus resembling digitalis, but that it is a much safer agent on account of its action being more steady and uniform.

To me its action seems compound, and as effecting its influence through both the organic and sympathetic systems of nerves. A dose of ergot given to a female will first affect the uterine tissue, especially if it be greatly enlarged from hypertrophy or gestation, or from any abnormal or foreign substances in its cavity. But it does not act till it has had time to enter the circulation and produce its power in this respect through stimulation of the nerves supplying that organ. Under other circumstances, its first effects seem to be in producing contractility of the tunics of the lesser arteries and veins. And this effect it doubtless brings about through its action on the vasomotor system of nerves. In this case it is not stimulation of these nerves, but depression, as it has been well understood for many years that the capillary vessels, under its long-continued use, fail to continue conveying their blood onward, and a stasis results, causing gangrene in parts most distant from the heart. This effect can only be brought about by paralyzing the vasomotor nerves, thus drawing from the capillary vessels their wonted stimulus needed to perform their function. Such paralyzing effects, however, can only be had after its long-continued or excessive use, hence does not render it an unsafe remedy. Another effect of the agent is on the organic nerve centres, producing giddiness, a feeling of constriction in the head, dilation of the pupils, dryness of the throat and mouth, and a slight confusion of ideation, while at the same time the number of cardiac contractions are evidently reduced. These effects lead me to believe that its effects are not nearly as much upon the cerebrum as upon the medulla oblongata and spinal cord, from which the nerves, which it seems most to affect, receive their principle or entire origin.

Since writing the above, I call to mind a case of hemorrhage from the bowels, in a case of typhoid fever. The patient, a young lady, seemed hopelessly affected with this fever, was greatly exhausted, and unable to be raised up when the passages from the bowels occurred. When the hemorrhage occurred, I gave ergot as freely as could be administered, hoping to check the life-blood that was fast ebbing away. The effect of the remedy was prompt and decided in accomplishing this end. The patient finally recovered. In a case of profuse hemoptysis, which occurred a few weeks since, ergot was used freely in combination with monsels salt. It evidently had a strong hemostatic effect over the case, but a faithful trial with it did not completely arrest the hemorrhage, which seemed finally to be completed by plumbi acet. and pulv. opii in very free doses. This last is the only case of failure of its hemostatic properties, and here the agent was very beneficial in moderating the amount of blood discharged.

